# Utility of surgical tumor resection for normal-pressure hydrocephalus associated with spinal schwannoma in the cauda equina: A case report and review of the literature

**DOI:** 10.1016/j.ijscr.2022.107575

**Published:** 2022-08-31

**Authors:** Kentaro Murayama, Seiji Shigekawa, Akihiro Inoue, Mashio Taniwaki, Riko Kitazawa, Takeharu Kunieda

**Affiliations:** a Department of Neurosurgery, Ehime University School of Medicine, 454 Shitsukawa, Toon, Ehime 791-0295, Japan; b Division of Diagnostic Pathology, Ehime University Hospital, 454 Shitsukawa, Toon, Ehime 791-0295, Japan

**Keywords:** CSF, cerebrospinal fluid, CT, computed tomography, Gd, gadolinium, MMSE, Mini-Mental State Examination, MRI, magnetic resonance imaging, NPH, normal-pressure hydrocephalus, Schwannoma in the cauda equina, Normal pressure hydrocephalus, Surgical tumor resection, Successful outcome

## Abstract

**Introduction and importance:**

Generally, schwannoma increases the concentration of protein in cerebrospinal fluid (CSF) and causes normal-pressure hydrocephalus (NPH) due to absorption disorders of CSF. Cases of NPH caused by spinal schwannoma in the cauda equina are very rare. Here, we report a case of spinal schwannoma-related NPH in the cauda equina successfully treated by surgical resection alone.

**Case presentation:**

A 78-year-old man presented with a 3-month history of gradually worsening memory disturbance. Neurological examination on admission showed dementia, hemiparesis of the left lower limb and gait disturbance. Computed tomography (CT) of the head revealed ventricular dilatation. CSF pressure was 150 mmH_2_O. CSF analysis showed a normal cell count and a highly elevated protein level (3842 mg/dL). Magnetic resonance imaging (MRI) of the lumbar spine demonstrated an enhanced intradural extramedullary mass in the cauda equina at the L3-L4 level. We suspected schwannoma causing NPH and tumor resection with posterior L3–4 laminectomy was performed as a priority. Marked recovery of cognitive dysfunction and gait disturbance was evident postoperatively, and CT 4 months later showed narrowing of the ventricles.

**Clinical discussion:**

If NPH due to spinal schwannoma is suspected as a result of lumbar puncture in a patient with dementia, confirmation of spinal schwannoma by lumbar MRI is absolutely necessary, and tumor resection alone may avoid unnecessary shunt placement.

**Conclusion:**

These findings suggest that if a spinal schwannoma located in the cauda equina causes symptoms due to NPH, removal of the tumor should be considered a priority.

## Highlights

• We report a case of NPH with spinal schwannoma in the cauda equina treated by surgical resection alone.
• Cases of NPH caused by spinal schwannoma in the cauda equina are very rare.
• These findings suggest that if a spinal schwannoma located in the cauda equina causes symptoms due to NPH, removal of the tumor should be considered a priority.
• Elderly patients presenting with NPH should have cerebrospinal fluid analysis and MRI screening of the spinal cord.
• This report will be invaluable in elucidating the pathogenesis of NPH with spinal neurinoma and its natural history.

## 1 Introduction and importance

Spinal schwannomas are common space-occupying disorders of the spinal canal. Schwannoma is well known to increase the concentration of protein in cerebrospinal fluid (CSF) and cause normal-pressure hydrocephalus (NPH) due to impaired absorption of CSF [1]. Several previous reports have discussed spinal schwannomas located in the cauda equina. However, cases of NPH caused by spinal schwannoma in the cauda equina are very rare and few cases have been described [1], [2], [3], [4], [5], [6], [7], [8]. Here, we report a case of NPH with spinal schwannoma in the cauda equina successfully treated by surgical resection alone. This work has been reported in line with the SCARE 2020 Criteria [9].

## 2 Case presentation

**Fig. 1:**
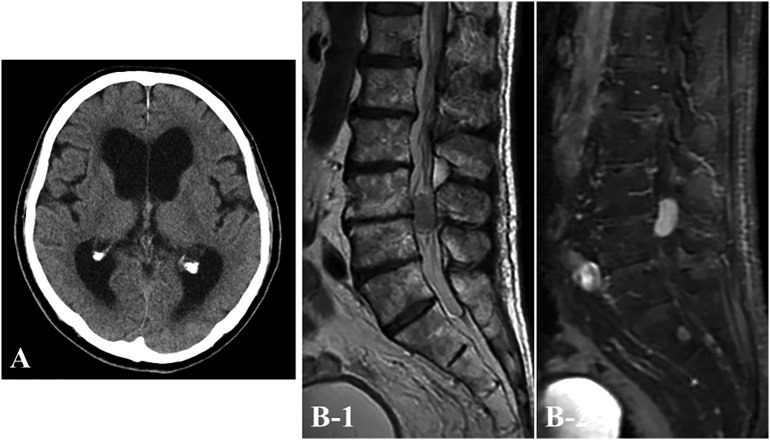
Preoperative computed tomography (CT) (A) shows a ventricular dilatation (Evans index, 0.4). Sagittal T2-weighted (B-1) and gadolinium (Gd)-enhanced T1-weighted (B-2) magnetic resonance imaging (MRI) reveal a homogeneously enhanced intradural extramedullary mass in the cauda equina at the L3-L4 level.

**Fig. 2:**
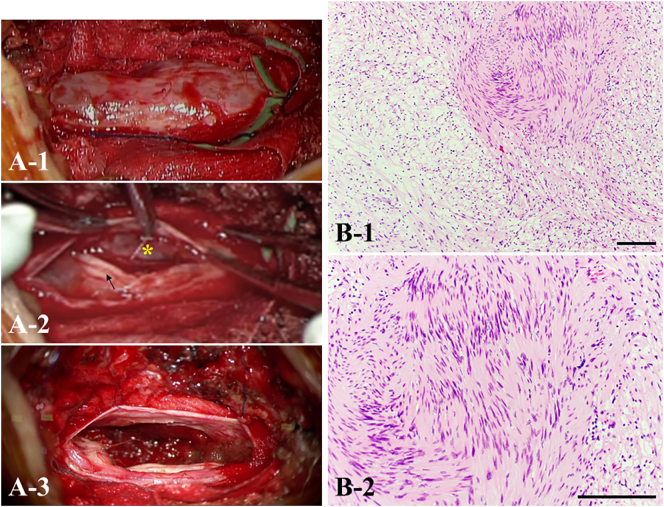
Intraoperative microscopic findings using a posterior approach. A-1) After posterior L3–4 laminectomy, a swollen dural sac is confirmed. A-2) After dissection of the arachnoid membrane, the tumor (yellow asterisk) is seen compressing the nerves of the cauda equina (black arrow). A-3) The tumor is grossly excised in its entirety. B) Histopathology of the resected lesion demonstrates mixed Antoni A and Antoni B components and a palisading pattern in accordance with typical features of benign schwannoma (hematoxylin and eosin staining). Magnification: B-1) × 100. Scale bar, 100 μm.; B-2) × 200. Scale bars, 100 μm. (For interpretation of the references to colour in this figure legend, the reader is referred to the web version of this article.)

**Fig. 3:**
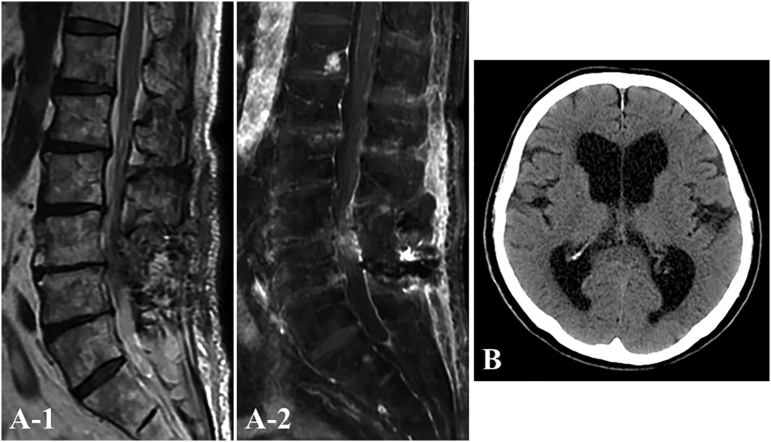
Postoperative sagittal T2-weighted (A-1) and Gd-enhanced T1-weighted (A-2) MRI after surgical resection show no residual tumor. CT after 4 months (B) demonstrates a slight reduction in ventricular size and clear depiction of the cerebral sulcus.

A 78-year-old man presented with a 3-month history of gradually worsening memory disturbance without headache. Neurological examination on admission showed dementia (Mini-Mental State Examination (MMSE): 21/30), hemiparesis of the left lower limb (manual muscle test: 4/V) and gait disturbance. On the other hands, there were not any neurological signs of urinary incontinence, retention and sexual disturbances were not recognized. Computed tomography (CT) of the head revealed ventricular dilatation (Evans index: 0.4) (Fig. 1A). Lumbar puncture was performed, and the CSF pressure was 150 mmH_2_O (institutional reference ranges: 60–180 mmH_2_O). CSF analysis showed a normal cell count (4.0 cells/μL) and a highly elevated level of protein (3842 mg/dL). Gadolinium (Gd)-enhanced magnetic resonance imaging (MRI) of the lumbar spine demonstrated a homogeneously enhanced intradural, extramedullary mass compressed the nerves at the cauda equina level (Fig. 1B). Based on MRI findings, preoperative differential diagnoses included spinal tumor, for example, schwannoma or meningioma. However, when the clinical course was taken into account, we suspected schwannoma and the highly elevated concentration of protein in the CSF was attributed to this schwannoma. We considered that this tumor was causing NPH and prioritized tumor resection. Under the general anesthesia, surgical resection by a posterior approach was performed in the prone position. Posterior L3–4 laminectomy was performed, the dural sac was opened, and cloudy arachnoid membrane was found. After dissecting the arachnoid membrane, the tumor was found to be compressing the nerves of the cauda equina. The tumor had apparently arisen from a root of the cauda equina, and electrical stimulations confirmed that the root was not a motor root of the limbs. Total removal (so called R0 resection) was performed, including the root (Fig. 2A). Histological examinations with hematoxylin and eosin staining demonstrated mixed Antoni A and Antoni B components and a palisading pattern, matching the typical features of benign schwannoma (Fig. 2B). No tumor cells were recognized in the nerves of the resected edge of the tumor. Postoperative MRI showed no residual tumor (Fig. 3A). The perioperative course was uneventful, and this intervention resulted in marked recovery of cognitive dysfunction (MMSE: 29/30) and gait disturbance with no sequelae. Lumbar puncture was performed 4 months postoperatively, and CSF analysis showed a decreased protein level (16 mg/dL) and a normal cell count (1.0 cells/μL). Postoperative CT after 4 months showed slight narrowing of the ventricles (Evans index: 0.35) and clear depiction of the cerebral sulcus (Fig. 3B), with no other symptoms or signs of NPH.

## 3 Clinical discussion

The frequency of NPH associated with spinal schwannoma, particularly occurred in the cauda equina is not yet clear. To the best of our knowledge, at the time of writing, only 22 cases of spinal schwannoma presenting with NPH have been reported previously [1], [8]. The mean age at diagnosis was 68.3 years (range, 45–82 years) and a female predominance was apparent (16/22 females; 72.7 %). Regarding the site of tumor origin, 72.7 % (16/22) of spinal tumors presenting with hydrocephalus occurred in the region under the thoracic spine [8]. Initial symptoms were most often a combination of neurological findings associated with NPH and spinal schwannoma (13/22; 59.1 %), but a minority of patients presented with symptoms of spinal schwannoma alone (2/22, 9.1 %) [8]. In our case, the patient was a 78-year-old man, and the site of tumor origin was in the cauda equina under the thoracic spine. In addition, the initial symptoms were a combination of dementia due to NPH and hemiparesis of the left lower extremity associated with spinal schwannoma.

Many hypotheses have been put forward concerning the mechanisms by which spinal schwannoma causes hydrocephalus [1], [2], [3], [4], [8]. The most widely accepted is that the increased protein in the CSF inhibits the absorption of spinal fluid in normal CSF circulation [1], [3]. Various mechanisms have been proposed to explain the elevated protein levels in the CSF in cases of spinal schwannoma. According to one theory, the schwannoma itself releases substances that cause elevation of protein levels in the CSF [1], [4]. Another hypothesis is that elevation of protein is mediated by immunological factors, because γ-immunoglobulin levels are elevated in the CFS of patients with spinal schwannoma associated with NPH [1], [2], [5]. In the present case, the protein level in the CSF was abnormally high (3842 mg/dL), but normalized after tumor resection along with improvement of the symptoms of NPH. We therefore consider that the tumor itself released some kinds of protein in this case.

Surgical procedures for NPH associated with spinal schwannoma can be proposed as either total tumor resection or shunt placement [1]. In 15 previously reported cases of NPH associated with spinal schwannoma, 11 cases (73.3 %) displayed complete resolution of NPH symptoms after tumor resection alone, suggesting the efficacy of total tumor resection for treating this pathology [1], [8]. However, four patients (26.7 %) required shunt surgery in addition to total tumor removal. In all 15 cases of NPH associated with spinal schwannomas, CSF protein levels were elevated (121–4073 mg/dl) [1], [8]. Average protein levels in CSF were 1338.4 mg/dL in the group of patients with tumor resection only and 2047.8 mg/dL in the group with shunt placement along with tumor resection. No significant differences in protein concentrations in CSF were evident between groups [1], [8]. The need for shunt surgery thus remains unclear, at least in terms of the effect on protein concentrations in CSF.

In most cases of NPH associated with spinal schwannoma, symptoms appeared to be relieved simply by surgical removal of the tumor [1], [8]. On the other hand, regarding tumor resection for cauda equina tumors, some authors have expressed concerns about possible neurological deficits that could result from resection of the root from which the cauda equina tumor arises [1], [5], [6]. However, tumor removal that includes the involved root does not always result in neurological deficits postoperatively. In the present case, CSF protein was abnormally high at the time of initial examination, at 3842 mg/dL. Removal of the spinal schwannoma resulted in a marked decrease in protein levels in the CSF and complete resolution of the symptoms of NPH and spinal schwannoma. Therefore, if NPH due to spinal schwannoma is suspected as a result of lumbar puncture in a patient with dementia, confirmation of spinal schwannoma by lumbar MRI is absolutely necessary, and tumor resection alone may avoid unnecessary shunt placement [1], [7]. Further experience with more cases is needed to clarify the safety and effectiveness of this treatment approach, and longer patient follow-up is required.

## 4 Conclusion

We have described a rare case of NPH associated with spinel schwannoma in the cauda equina in which successful treatment was achieved by surgical excision of the tumor only. NPH associated with spinal schwannoma occurring in the cauda equina is a rare but well-documented entity. Spinal schwannoma can cause NPH by disturbing the normal CSF circulation. In such cases, total tumor removal is often sufficient to resolve all symptoms induced by the tumor, even if accompanied by NPH. In addition, shunt placement may be best avoided, instead prioritizing resection of the spinal schwannoma. This report will be invaluable in elucidating the pathogenesis of NPH with spinal neurinoma and its natural history.

## Provenance and peer review

Not commissioned, externally peer-reviewed.

## Sources of funding

This research received no specific grant from any funding agency in the public, commercial, or not-for-profit sectors.

## Ethical approval

The clinical study of the above-mentioned case report was approved by the Ethics Committee for Clinical Research of Ehime University Hospital, and informed consent was obtained from the patient prior to initiating the study.

## Consent

Written informed consent was obtained from the patient for publication of this case report and accompanying images. A copy of the written consent is available for review by the Editor-in-Chief of this journal on request.

## Author contribution

Kentaro Murayama: writing the draft of this manuscript and clinical management; Seiji Shigekawa: surgical treatment and clinical management; Akihiro Inoue: clinical management, supervision, review and editing of this manuscript; Mashio Taniwaki: pathological diagnosis; Riko Kitazawa: pathological diagnosis and supervision; Takeharu Kunieda: review and editing of the manuscript.

## Registration of research studies

This paper is a case report and was therefore not required to be registered with any registry.

## Guarantor

Akihiro Inoue.

## Declaration of competing interest

None of the authors have any commercial or financial involvement in connection with this study that represents or appears to represent any conflicts of interest.
